# Cardiopulmonary bypass in the newborn: effects of circulatory cell-free hemoglobin and hyperoxia evaluated in a novel rat pup model

**DOI:** 10.1186/s40635-017-0153-2

**Published:** 2017-10-04

**Authors:** Åsa Jungner, Suvi Vallius, Matteo Bruschettini, Olga Romantsik, Magnus Gram, David Ley

**Affiliations:** 1 Department of Clinical Sciences Lund, Pediatrics, Skane University Hospital, Lund University, Lund, Sweden; 2 Department of Clinical Sciences Lund, Pediatric Surgery and Neonatal Care, Skane University Hospital, Lund University, Lund, Sweden; 3 Department of Clinical Sciences Lund, Infection Medicine, Lund University, Lund, Sweden; 4 Pediatric Intensive Care Unit (BIVA), Skane University Hospital, Lund, Sweden

**Keywords:** Congenital heart disease, Cardiopulmonary bypass, Cell-free hemoglobin, Hyperoxia, Neonate, Animal model, Inflammation, Oxidative stress, Haptoglobin, Hemopexin

## Abstract

**Background:**

Infants with congenital heart defects (CHD) are at risk for white matter brain injury. This novel rat pup model characterizes the systemic effects of intravasal cell-free hemoglobin and hyperoxia, hypothesizing that immature endogenous scavenging systems relate to increased vulnerability to conditions present during cardiopulmonary bypass (CPB).

**Methods:**

Plasma pharmacokinetics of cell-free human hemoglobin (Hb) was determined after intraperitoneal (i.p.) administration in postnatal day 6 (P6) rat pups. Cell-free hemoglobin degradation, scavenger- and oxidative stress responses in altered oxygen environments were evaluated in P6 rat pups exposed to i.p. cell-free Hb or vehicle and subjected to hyperoxia or normoxia for 24 h. Plasma and liver were analyzed for free heme, haptoglobin, hemopexin, heme-oxygenase 1, and 8-OHdG at 3–120 h post-injection. Baseline scavenging properties were evaluated in P0-P12 rat pups.

**Results:**

Cell-free Hb displayed peak plasma concentrations of 3.6 ± 0.5 mg/mL (mean ± SD) at 3 h post-administration. Animals exposed to cell-free Hb demonstrated a 30-fold increase in plasma haptoglobin and a decrease in plasma hemopexin to 1/6 of concentrations observed in pups exposed to vehicle. Exposure to cell-free Hb and hyperoxia mediated increased plasma concentrations of free heme (72.7 ± 19.5 μM, mean ± SD) compared to exposure to cell-free Hb and normoxia (49.3 ± 13.1 μM) at 3 h, and an elevated hepatic mRNA expression of heme-oxygenase 1. mRNA expression of haptoglobin and hemopexin was increased in animals exposed to hemoglobin with a mitigated response in pups exposed to hemoglobin and hyperoxia. Animals exposed to hyperoxia displayed an increase in hepatic transcription of scavenger proteins at 24 h. Combined exposure to cell-free Hb and hyperoxia mediated an increased DNA-oxidation at 6 h, whereas all insults conveyed a decrease in DNA-oxidation at 120 h.

**Conclusions:**

In this study, we present a novel rat pup model with scavenging characteristics and brain maturation similar to newborns with CHD. We have confirmed a distinct scavenger response after exposure to systemic cell-free hemoglobin. We have indications of an accelerated metabolism of cell-free Hb and of an altered transcription of scavenger proteins in a hyperoxic environment. We believe that this model will prove valuable in future delineation of inflammatory and oxidative end-organ damage following CPB.

**Electronic supplementary material:**

The online version of this article (10.1186/s40635-017-0153-2) contains supplementary material, which is available to authorized users.

## Background

The management of congenital heart defects (CHD) has seen remarkable improvements in mortality rates over the last decades. With increasing survival, it has been apparent that newborn infants with CHD requiring early correction on cardiopulmonary bypass (CPB) are at an increased risk for impaired long-term neurodevelopmental outcome with diffuse white matter brain injury as the predominant neuro-radiological correlate [1]. In a recent scientific statement by the American Heart Association, survival rate was recognized as above 80% in newborns with CHD requiring surgery on CPB, whereas 30–50% of long-time survivors exhibit varying degrees of impaired neurodevelopmental outcome [2].

CPB-assisted open-heart surgery results in a powerful systemic inflammatory response and oxidative stress [3–5]. Initiating events are likely to be multifactorial with ischemia-reperfusion and contact with non-endothelial surfaces as often-cited initiators [6]. Circulation of blood in the CPB circuit is known to result in mechanically induced hemolysis with increased levels of cell-free (extracellular) hemoglobin (Hb), which have been suggested as causally involved in organ dysfunction and physiological disturbances following CPB [7–9].

Cell-free Hb is a highly reactive molecule that is rapidly oxidized from ferrous (Fe^2+^, denoted oxyHb) to ferric (Fe^3+^, denoted metHb) Hb while producing reactive oxygen species and reducing the bioavailability of NO [10, 11]. Ferric Hb readily releases free heme, a redox reactive molecule with a capacity of inflicting oxidative damage to lipids, proteins, and DNA, and also of toxic cytolytic effects by intercalation into cell membranes [12, 13]. Moreover, free heme has been suggested as a TLR4-ligand, indicating an inflammation-inducing propensity [14]. The impact of cell-free Hb on the inflammatory process and oxidative stress following CPB is not well characterized; neither are the temporal aspects of cell-free Hb metabolism in a hyperoxic environment.

The major human endogenous scavenger systems of cell-free Hb and its metabolite heme are haptoglobin (Hp) and hemopexin (Hpx) [15–17]. These have been well characterized as abundant and efficient in the human adult and in mature animal models. The newborn infant has, in comparison, low endogenous levels of these plasma proteins [18, 19], suggesting a heightened vulnerability to the potentially toxic effects of cell-free Hb and its down-stream metabolites.

Oxygen is a toxic molecule when in surplus. Previous research from our and other groups show that hyperoxemia per se or during reperfusion following hypoxia-ischemia plays a causal role in end-organ damage [20, 21]. Clinical studies performed during pediatric CPB suggest that levels of oxidation, inflammation, and end-organ damage markers are dependent on whether supra-normal or normal levels of oxygen are used when on CPB in both cyanotic and non-cyanotic patients [22, 23]. Cyanotic patients appear to fare worse in terms of oxidative damage than non-cyanotic patients on CPB even when normoxia is applied [24].

The combined effects of circulating cell-free Hb and hyperoxemia have, to the best of our knowledge, not previously been studied in either immature or mature in vivo model systems. Both insults are of clinical relevance during CPB in the newborn period and occur simultaneously. We hypothesized that the additive effects of cell-free Hb and hyperoxemia would result in a potentiated induction of harmful mechanisms related to cell-free hemoglobin toxicity and thereby lead to aggravated tissue damage. To this end, we developed a novel immature rat pup model aiming to characterize the systemic effects of intravasal cell-free Hb and hyperoxemia. Postnatal day 6 (P6) rat pups were exposed to a combined insult of (1) systemic cell-free Hb or vehicle solution administered intraperitoneally (i.p.) and/or (2) a hyperoxic or normoxic environment and examined for endogenous scavenger and oxidative stress responses. Here, we present data displaying our novel rat pup model as a valuable tool for investigation of systemic hemolysis and concomitant hyperoxemia in an immature physiology with a potential value for future studies on end-organ damage.

## Methods

### Animals

The experiments were performed on a total of 319 Wistar rat pups (Scanbur Research A/S Karslunde, Denmark) from 45 litters. All rat pups were nursed and fed by lactating dams throughout the study. Animals were handled according to Swedish animal welfare legislation. The research protocol was approved by the Swedish Animal Ethics Committee in Lund, permit nr M48-14.

### Preparation of human cell-free Hb

Human cell-free Hb was purified as previously described [25] from human adult red blood cells obtained from the blood center in Lund (Sweden). The Hb concentration was quantified using Plasma/Low Hb (Hemocue, Ängelholm, Sweden). The Hb was dissolved in Ringer’s Acetate (Baxter, Deerfield, Ill, USA) and purified from endotoxin contamination using EndoTrap as described by the manufacturer (Hyglos GmbH, Germany). The absolute purity of Hb from contamination with endotoxin was determined by QCL-1000™ Endpoint Chromogenic LAL Assay as described by the manufacturer (Lonza, Switzerland).

### Experimental setup 1: pharmacokinetics of cell-free human Hb following i.p. administration

**Fig. 1:**
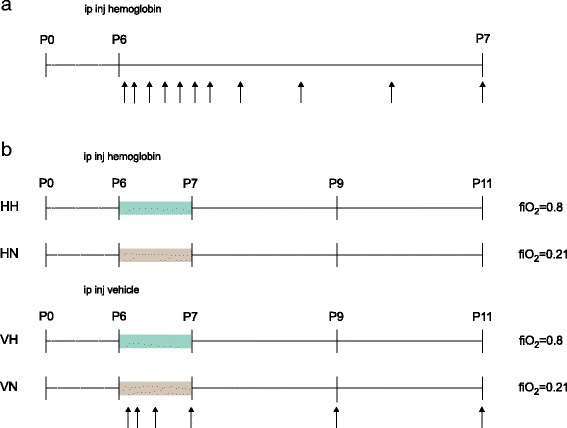
Overall study design. **a** Experimental setup 1. P6 rats were injected i.p. with cell-free human Hb. Blood was sampled as indicated by arrows. *n* = 5–7 at each time point. **b** Experimental setup 2. P6 rat pups were randomized into four groups, *n* = 5–11 in each group: (1) i.p. cell-free Hb injection and 24 h hyperoxia (HH), (2) i.p. cell-free Hb injection and 24 h normoxia (HN), (3) i.p. vehicle injection and 24 h hyperoxia (VH), and (4) i.p. vehicle injection and 24 h normoxia (VN). The blood and liver tissue were collected as indicated by arrows

Litter-mixed P6 Wistar rat pups were sedated with isoflurane in room air and injected i.p. with a weight-based dose of endotoxin-free cell-free human Hb purified as described above or with corresponding volume of vehicle (Ringer’s Acetate). Dose calculations were made assuming (1) a plasma volume of 40 ml/kg, (2) an estimated peritoneal transfer of 1/10 of administered dose [26], and (3) an attempted plasma concentration of 5 mg/ml, resulting in administered doses of 2 g/kg of cell-free hemoglobin from a solution containing 200 mg/mL. Animals (*n* = 5–7 at respective time point) were sacrificed by decapitation at 0.5, 1, 2, 3, 4, 5, 6, 8, 12, 18, and 24 h following i.p. injection. The study design is illustrated in Fig. 1a. Blood samples were collected in sodium-citrate tubes (MiniCollect 450413, Greiner Bio-One, Kremsmünster, Austria); plasma was separated by centrifugation at 2000×*g* for 10 min and stored at − 80 °C until further use.

### Experimental setup 2: systemic exposure to cell-free human Hb and hyperoxemia

Litter-mixed P6 Wistar rat pups were randomized into one of the following groups: (1) rat pups receiving i.p. cell-free Hb injections and exposure to hyperoxia (group denoted HH), (2) rat pups receiving i.p. cell-free Hb injections and exposure to normoxia (group denoted HN), (3) rat pups receiving i.p. vehicle injections and exposure to hyperoxia (group denoted VH), and (4) rat pups receiving i.p. vehicle injections and exposure to normoxia (group denoted VN). Overall study design including the respective study groups is illustrated in Fig. 1b.

The experimental procedure was carried out as follows: rat pups were sedated with isoflurane in room air and injected i.p. with a weight-based dose of endotoxin-free cell-free human Hb purified as described above or with corresponding volume of vehicle (Ringer’s Acetate). Dose calculations of cell-free Hb were made as described above. After recovery, pups were returned to a lactating dam and placed in neonatal incubator (Dräger 8000, Drägerwerk AG, Lübeck, Germany) with exposure to either a hyperoxic (fiO2 0.8) or a normoxic (fiO2 0.21) condition for 24 h. Oxygen concentrations within the incubators were monitored by the in-built monitoring system of the incubator and crosschecked using a portable oxygen monitor (MX300-I, Teledyne Analytical Instruments, Los Angeles, CA, USA). Hyperoxemia was confirmed by blood gas analysis of blood samples obtained by decapitation after 45 min exposure to hyperoxia. Temperature in incubators was kept constant at 28 °C. After the 24 h exposure to hyperoxia or normoxia, animals were housed in room air and room temperature until termination.

Animals were sacrificed by decapitation at 3, 6, 12, 24, 72, and 120 h post cell-free Hb or vehicle administration. Blood samples were collected in sodium-citrate tubes (Greiner Bio-One); plasma was separated by centrifugation at 2000×*g* for 10 min and stored at − 80 °C until further use. Liver tissue was snap-frozen on dry ice immediately after excision and stored at − 80 °C until further use.

### Analysis of cell-free Hb

Plasma concentration of cell-free Hb was evaluated using Plasma/Low Hb (Hemocue, Ängelholm, Sweden) and human Hb ELISA according to instructions from the manufacturer (Genway Biotech Inc., San Diego, Ca, USA). Ability to discriminate between cell-free rat Hb and cell-free human Hb was confirmed by analysis of plasma from animals not subjected to i.p. injection of human cell-free Hb.

### Analysis of in vivo Hb-degradation

Evaluation of in vivo Hb-degradation was performed by analyzing the presence of free heme using the Heme Colorimetric Assay Kit (BioVision, Milpitas, CA, USA) in plasma of animals subjected to varied oxygen environments.

### Analysis of Hb-scavengers in plasma

Plasma levels of the endogenous Hb-scavengers Hp and Hpx were determined using rat Hp and Hpx ELISA assays as described by the manufacturer (Genway Biotech Inc.). Baseline plasma levels of Hp and Hpx were determined in rat pups not subjected to any intervention before decapitation at P0, P2, P4, P5, P6, P7, P8, and P12.

### RNA isolation and real-time PCR

Expressions of Hp, Hpx, and heme oxygenase 1 (HMOX1) mRNA respectively were analyzed in liver tissue of animals terminated at 3, 12, and 24 h. Total RNA was isolated from liver tissue using NucleoSpin® RNA/protein (Marchery-Nagel, Duren, Germany) and RNeasy Mini Kit supplied by Qiagen (Germantown, MD, USA). The OD ratio (optical density at 260 nm/280 nm) of RNA was always higher than 1.95. Reverse transcription was performed according to the manufacturer on 1.0 μg total RNA using RT^2^ First Strand Kit (Qiagen). RT^2^ qPCR Primer Assays (from Qiagen) were used to quantify the mRNA expression of Hp, Hpx, and HMOX1. Data were normalized to Ribosomal protein, large, P1 (Rplp1; Qiagen). The fold change values were calculated by normalizing against the mean of the VN group at all time points.

### DNA-oxidation

Oxidative DNA damage in plasma samples was evaluated using the OxiSelect™ Oxidative DNA damage ELISA Kit as described by the manufacturer (Cell Biolabs Inc., San Diego, CA, USA) at 3, 6, 12, 24, and 120 h.

### Statistics

All statistics were performed using GraphPad Prism7 software (La Jolla, CA, USA). Results are presented as mean ± standard deviation unless otherwise stated. Comparisons between groups were made assuming parametric data using Student’s *t* test for pairwise comparisons or one-way ANOVA with post hoc Tukey’s test for multiple group comparisons at respective time point. *p* values < 0.05 were considered significant.

## Results

### Pharmacokinetics of cell-free human Hb

**Fig. 2:**
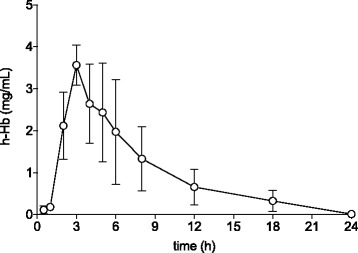
Human cell-free Hb pharmacokinetics (mean ± SD) following i.p. administration. *n* = 5–7 at each time point

The pharmacokinetics of cell-free human Hb was analyzed in plasma at 0–24 h following i.p. administration. A rapid circulatory uptake of cell-free human Hb was observed, reaching a maximum level of 3.6 ± 0.5 (mean ± SD) mg/ml at 3 h after injection followed by an elimination with no circulatory levels of cell-free human Hb detected at 24 h post-injection (Fig. 2).

### Hb-degradation in vivo

**Fig. 3:**
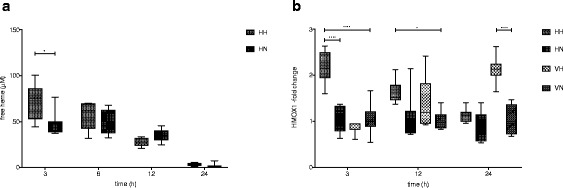
Hb-metabolism in vivo. **a** Plasma concentrations of free heme 3–24 h post i.p. administration of cell-free human Hb. *n* = 5–11 in all groups. **b** Liver mRNA expression of HMOX1 at 3–24 h post i.p. cell-free Hb injection. Fold change was calculated against VN-mean at each time point. *n* = 5–7 in all groups. Results are presented as *box-and-whisker plots* with min and max values marked

Hb degradation in vivo was evaluated by analyzing the levels of the Hb-metabolite free heme in plasma at 3–24 h post i.p. administration of cell-free human Hb. Free heme was only detectable in plasma of animals exposed to cell-free Hb (HH + HN). Maximal concentrations were reached at 3 h post i.p. administration and declined thereafter with no detectable plasma concentrations at 24 h (Fig. 3a). Animals exposed to the combined insult (HH) had a significantly higher concentration of plasma heme at 3 h (72.7 ± 19.5 μM (mean ± SD)) as compared to those exposed to Hb and normoxia (HN; 49.3 ± 13.1 μM (mean ± SD), *p* = 0.02).

The hepatic mRNA expression of the heme-degrading protein HMOX1 was analyzed at 3–24 h post i.p. injection and was found to be significantly increased at 3 h in the HH group as compared to the HN group (*p* < 0.0001) and the VN group (*p* < 0.0001) (Fig 3b). At 12 h, the HH group had a sustained elevated expression of HMOX1 compared to the VN group (*p* = 0.04). A significantly increased HMOX1 mRNA expression was found in the group exposed to hyperoxia (VH) at 24 h post i.p. injection as compared to the VN group (*p* < 0.0001)(Fig. 3b).

### Endogenous Hb-scavenging systems

**Fig. 4:**
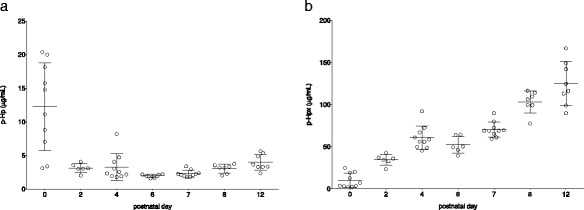
Baseline Hb- and heme-scavenging systems. Hp (**a**) and Hpx (**b**) (mean ± SD) were analyzed from P0-P12. *n* = 6–10 at all time points

In order to establish an understanding of the endogenous circulatory Hb- and heme-scavenging systems, baseline levels of Hp (cell-free Hb scavenger) and Hpx (heme scavenger) were analyzed in rat pups with a postnatal age of P0-P12. The mean plasma concentrations of Hp were low at all postnatal ages with a nadir at P6 and a subsequent slow rise with increasing postnatal age (Fig. 4a). Hp concentrations at P0 were highly scattered and displayed fivefold maximum levels compared to concentrations in P2 rats. Hpx displayed the lowest baseline mean plasma concentration at P0 and a linear increase with increasing postnatal age (Fig. 4b).

**Fig. 5:**
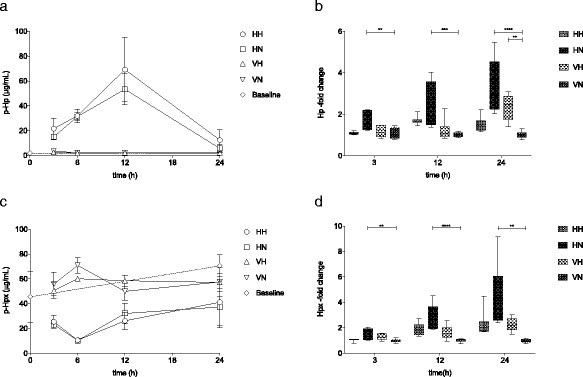
Hb- and heme-scavenging systems following exposure to cell-free human Hb and/or hyperoxia. Plasma concentrations (**a**, **c**) and mRNA expression in liver (**b**, **d**) of Hp (**a**, **b**) and Hpx (**c**, **d**) at 3–24 h following i.p. injection. Fold change mRNA expression was calculated against VN-mean at each time point. *n* = 5–18 in all groups. Results are presented as ﻿mean ± SD for **a** and **c** and as *box-and-whisker plots* with min and max values marked for **b** and **d**

Following i.p. administration of cell-free human Hb (HH and HN groups), plasma concentrations of Hp increased rapidly reaching a 30-fold increase as compared to baseline levels at 12 h (Fig. 5a). Following the peak levels at 12 h, a decline towards baseline was observed at 24 h post i.p. injection. Plasma levels of Hp in groups not exposed to cell-free human Hb (VH and VN) corresponded to baseline levels at all time points.

Following i.p. cell-free human Hb injection (HH and HN groups), plasma concentrations of Hpx were decreased to 1/6 of levels observed in animals injected with vehicle (VN and VH groups) at 6 h post i.p. injection. Plasma levels of Hpx in groups not exposed to cell-free human Hb (VH and VN) corresponded to baseline levels at all time points (Fig. 5c).

Of note, exposure to pure hyperoxia (VH) caused no discernible change in plasma-concentrations of either Hp or Hpx at any time point. Hp and Hpx concentrations in plasma did not differ in the group exposed to the combined insult (HH) as compared to those exposed to cell-free Hb only (HN).

The hepatic mRNA expression of Hp and Hpx was evaluated at 3–24 h post i.p. cell-free human Hb administration. Hp expression was increased at 3 h (*p* = 0.006), 12 h (*p* = 0.0001), and 24 h (*p* < 0.0001) in the group subjected to cell-free human Hb and normoxia (HN) compared to the vehicle normoxia (VN) group (Fig. 5b). The group subjected to cell-free human Hb and hyperoxia (HH) had an increased expression of Hp mRNA at 12 and 24 h compared to the VN group, but the increase did not reach statistical significance.

Hpx mRNA expression was significantly increased at 3 h (*p* = 0.003), 12 h (*p* < 0.0001), and 24 h (*p* = 0.005) in the group subjected to cell-free Hb and normoxia (HN) compared to the VN group (Fig. 5d). The group subjected to cell-free Hb and hyperoxia (HH) had an increased expression of Hpx mRNA at 12 and 24 h compared to the VN group, but the increase did not reach statistical significance.

Notably, mRNA expression of Hp increased significantly in the group subjected to hyperoxia only (VH) as compared to the VN group at 24 h (*p* = 0.008). An increased expression of Hpx mRNA was seen in the VH group compared to the VN group at 24 h, but the increase did not reach statistical significance (Fig. 5b, d).

### Systemic oxidative stress

**Fig. 6:**
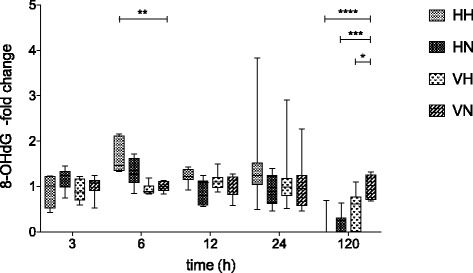
DNA-oxidation in plasma. Systemic oxidative stress measured as DNA-oxidation (8-OHdG) in plasma at 3–120 h post i.p. injection. Fold change was calculated against VN-mean at each time point. *n* = 5–18 in all groups. Results are presented as *box-and-whisker plots* with min and max values marked

The level of systemic oxidative stress measured as DNA-oxidation (8-OHdG) was evaluated in plasma at 3–120 h post i.p. injection. Animals exposed to the combined insult of cell-free human Hb and hyperoxia (HH) showed a significant increase in plasma DNA-oxidation as compared to animals injected with vehicle and subjected to normoxia (VN) at 6 h (*p* = 0.02). At 120 h, all groups subjected to any insult (HH, HN, and VH) displayed significantly lower values of DNA-oxidation as compared to the VN group (Fig. 6).

The dataset supporting our results can be found as an Additional file 1.

## Discussion

Hyperoxemia and elevated levels of systemic cell-free Hb are insults with an increased potential for toxicity in an immature system compared to an adult counterpart. Both term and preterm neonates have a proven susceptibility to oxygen toxicity [27] and their endogenous Hb-scavenger resources are scarce [18, 19]. The interplay between systemic inflammation and hyperoxic damage is complex and possibly dependent on the temporal relation of events [28–30]. In this study, we present a rat pup model with a progressive increase in circulatory cell-free Hb and simultaneous hyperoxemia that accurately mimics the temporal relationship between induced hemolysis and exposure to hyperoxia during CPB.

Rat pups have been used extensively for exploring mechanisms of neonatal brain damage. Brain maturity in a P7 rat pup is considered similar to that of a newborn infant born at term [31]. Although infants with congenital cardiac defects are mainly born at term, their brain maturity has been shown to correspond to that of a slightly preterm neonate [32, 33], justifying the choice of the P6 rat pup as model animal. We found that baseline levels of the endogenous Hb-scavenger systems, Hp and Hpx, were low at P6 in the rat pup, which corresponds to the very low circulating levels observed in the newborn human infant [18, 19].

Intraperitoneal administration of cell-free Hb could be performed safely without local short- or long-term negative effects related to i.p. deposition. A reproducible and predictable uptake of cell-free human Hb into the systemic circulation was achieved, and the resulting systemic levels of cell-free Hb were similar to those reported in children on CPB [34]. Attempts to further increase the plasma levels by increased i.p. deposition were not successful due to limitations in volume and viscosity of the i.p. injectate and possibly also due to renal excretion of cell-free Hb.

An important advantage of administering human cell-free Hb was that it enabled us to discriminate circulatory uptake of cell-free Hb from sampling-derived hemolysis. A possible disadvantage of this setting would be the risk of introduction of a protein foreign to the species, which in itself might provoke inflammatory and immunological reactions. However, as the three-dimensional structure of Hb in vertebrates is highly conserved throughout evolution [35], we do not think that the cross-species injection introduces a significant source of error in the interpretation of the results.

In our study, we found an increase in free heme at 3 h concurrent with the maximum levels of plasma cell-free Hb. The difference in heme levels in relation to different oxygen environments at 3 h is interesting and indicates an accelerated Hb metabolism in a hyperoxic environment. The early rise in HMOX1 transcription is in accordance with this speculation. We interpret the lack of difference in heme-levels at 6 h as a consequence of the decreased absolute levels of cell-free Hb and, thus, a relative lack of substrate bringing free heme levels under the threshold of Hpx scavenging capacity.

Haptoglobin in plasma increased to a maximum 30-fold rise at 12 h in Hb-injected animals compared to animals injected with vehicle. This finding is unexpected since exposure to cell-free Hb is commonly associated with a decrease in Hp-levels as the Hb-Hp complex is cleared from plasma. We considered the possibility that the many-fold surge in plasma concentrations of Hp represents an acute phase response as Hp is a well-recognized acute phase reactant in rodents [36], and indeed, we observed an increased transcription of Hp in liver tissue in both Hb-exposed groups at 12 h that parallels maximal plasma concentrations. We did not find an altered glycosylation pattern of Hp as an indication of neutrophil-derived Hp-release into systemic circulation [37].

Hemopexin levels in Hb-injected animals were approximately halved within 6 h of Hb injection in comparison with vehicle-injected animals signifying endocytosis and scavenging of the heme-Hpx complex. At 24 h Hpx-levels were almost comparable to those in animals injected with vehicle and to baseline animals. This result is in line with the observed rapid decrease in levels of free heme. Hpx transcription in liver was increased at 3, 12, and 24 h in animals injected with cell-free Hb, presumably as an endogenous response to consumption.

In mRNA analyses of Hp and Hpx, the translational response was mitigated in the HH group as compared to the HN group. The attenuating effect of hyperoxia on hepatic regulation of Hp and Hpx was an unexpected finding. There are numerous reports of transcriptional modifications in different organs in the presence of hyperoxia [38, 39], but to the best of our knowledge, none of them encompass hepatic transcription of hemoglobin-scavenging genes. As of now, we do not have any immediate explanation of this finding. Another interesting observation in our model was the increase in hepatic transcription of all measured Hb- and heme-scavenging proteins in response to hyperoxemia only (VH) at 24 h. The increased transcription indicates broader scavenging properties than previously thought.

The evidence of systemic toxicity and oxidative stress was supported by the pattern of plasma DNA-oxidation found both with singular and combined insults. We believe that the increase in DNA-oxidation at 6 h supports the idea that combined exposure to cell-free Hb and hyperoxia results in additive oxidative stress in an immature physiology. The significant decrease in DNA-oxidation at 120 h in all groups exposed to any insult (HH, HN, and VH) may be interpreted as an activation of DNA repair mechanisms as a result of induced oxidative stress. Whether this oxidative stress translates into clinically significant end organ damage remains to be investigated.

A possible limitation of using rats for evaluation of cell-free Hb toxicity is the rat’s ability to produce ascorbic acid, a significant antioxidant as shown in adult rats [40, 41]. Humans rely on dietary supplement of ascorbic acid as do guinea pigs, the only rodent with a similar incapability of endogenous production. However, as we aimed to mimic a neonatal physiology, guinea pigs were not a preferred alternative due to their accelerated brain maturity at birth [42]. We analyzed plasma levels of ascorbic acid and mRNA expression of the GULO-enzyme in liver tissue (data not shown), but in neither analyses did we obtain reproducible results. We acknowledge the fact that our immature rat model probably has an increased inherent capacity of antioxidant defenses compared to humans, and may only speculate upon possible consequences.

## Conclusions

In this manuscript, we present data of a novel rat pup model mimicking two potentially injurious aspects of CPB in newborn infants. We have shown that i.p. injection of cell-free Hb is a feasible way of achieving a predictable and reproducible level of systemic cell-free Hb with a consistent plasma scavenger response. We have established baseline Hb-scavenging properties similar to those of the human newborn. We have indications of an accelerated metabolism of plasma cell-free Hb and of an altered transcriptional regulation of scavenging proteins in a hyperoxic environment. Our results suggest an activation of DNA repair mechanisms following all insults, singular or combined. We believe that the presented animal model is valuable and well suited for further investigations on organ-specific consequences of systemic hemolysis and concomitant hyperoxemia in an immature physiology.

## Additional file

Additional file 1: Dataset supporting our results. (XLSX 81 kb)

## Abbreviations

CHD: Congenital heart defect
CPB: Cardiopulmonary bypass
Hb: Hemoglobin
HMOX-1: Heme oxygenase 1
Hp: Haptoglobin
Hpx: Hemopexin
i.p.: Intraperitoneal
P: Postnatal day
